# Mucins, gut microbiota, and postbiotics role in colorectal cancer

**DOI:** 10.1080/19490976.2021.1974795

**Published:** 2021-09-29

**Authors:** Ramesh Pothuraju, Sanjib Chaudhary, Satyanarayana Rachagani, Sukhwinder Kaur, Hemant K. Roy, Michael Bouvet, Surinder K. Batra

**Affiliations:** a Department of Biochemistry and Molecular Biology, University of Nebraska Medical Center, Omaha, NE, USA; b Department of Medicine, Baylor College of Medicine, Houston, TX, USA; c Division of Surgical Oncology, Department of Surgery, University of California San Diego, La Jolla, CA, USA; d VA San Diego Healthcare System, San Diego, CA, USA; e Fred and Pamela Buffett Cancer Center, University of Nebraska Medical Center, Omaha, NE, USA; f Eppley Institute for Research in Cancer and Allied Diseases, University of Nebraska Medical Center, Omaha, NE, USA

**Keywords:** Mucins, gut microbiota, prebiotics, probiotics, postbiotics, colorectal cancer

## Abstract

An imbalance in the crosstalk between the host and gut microbiota affects the intestinal barrier function, which results in inflammatory diseases and colorectal cancer. The colon epithelium protects itself from a harsh environment and various pathogenic organisms by forming a double mucus layer, primarily comprising mucins. Recent studies are focusing on how dietary patterns alter the gut microbiota composition, which in turn regulates mucin expression and maintains the intestinal layers. In addition, modulation of gut microbiota by microbiotic therapy (involving fecal microbiota transplantation) has emerged as a significant factor in the pathologies associated with dysbiosis. Therefore, proper communication between host and gut microbiota *via* different dietary patterns (prebiotics and probiotics) is needed to maintain mucus composition, mucin synthesis, and regulation. Here, we review how the interactions between diet and gut microbiota and bacterial metabolites (*postbiotics*) regulate mucus layer functionalities and mucin expression in human health and disease.

## Introduction

Various bacteria, archaea, viruses, phages, yeast, protozoa, and fungi are present in the human body and are known as microbiota. These organisms are located in the skin, lungs, gut, vaginal, and urinal tracts of the body and form a symbiotic relationship because they associate with almost all human cells.^1,2^ Microbiota within a specific microenvironment such as the gut is named “*gut microbiota*”.^3^ Around 10^11^ bacterial cells and 10 million bacterial genes (*gut microbiome*) in the large intestine contribute to a healthy metabolic status in the host. In the large intestine, the microbial population is very dense in the lumen and sparse near epithelial cells, though some bacterial species adhere and reside in the mucus layer.^4^ In germ-free mice, mucus layer formation and mucin glycosylation pattern were distinct from conventionally raised mice due to the absence of microbiota.^5^ Additionally, microbial products such as lipopolysaccharides and peptidoglycans in conventional mice stimulate mucus secretion.^6^ Thus, the dynamic interplay between the gut microbiota and the host is important to maintain the intestinal mucus layer.

**Figure 1.**
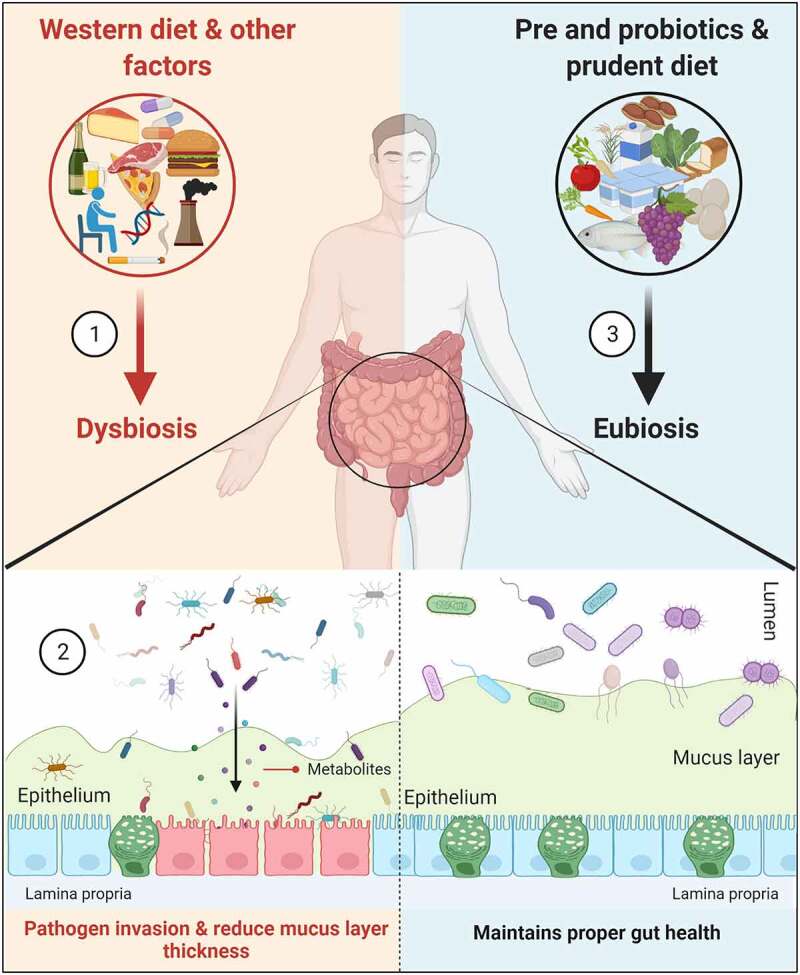
Amelioration of gut microbiota and mucins. (1). Consumption of a Western-style diet (rich in high-fat calories and low in fiber) and other factors mediates microbiota alterations (dysbiosis) in the colon. (2). This altered microbiota, along with their metabolites, are responsible for increasing the intestinal permeability and reducing the mucus layer thickness by decreasing MUC2 expression; the result is the invasion of pathogens into the epithelium. (3). Administration of pre- (GOS and FOS) and probiotics (majorly bifidobacteria and lactobacilli) and prudent diet improves the intestinal layer functionalities and maintains proper gut health (eubiosis)

Diet, genetics, drugs, environmental factors, and the state of disease mediate *gut dysbiosis*, which results in the infiltration of bacterial components from the lumen to the mucus layer (Figure 1). Individuals with low bacterial richness are more prone to metabolic disorders as compared to those with high bacterial richness.^7^ In healthy individuals, the gut microbiota is linked with higher mucus layer thickness, which results in improved glucose metabolism by the short-chain fatty acids (SCFAs). In contrast, thinner mucus is linked with alterations in gut microbiota in disease,^8,9^ though the underlying mechanism and which microbiota are involved in forming the mucus layer are not known. Research on the role of microbiota in metabolic diseases waned in the early 2000s; however, more recent studies on the interdependence of gut microbiota in inflammatory bowel diseases (Ulcerative colitis, UC and Crohn’s disease, CD) and colorectal cancer (CRC),^10^ revived interest in the field. For instance, fecal metagenomics analysis revealed several bacterial species (*Bacteroides fragilis, Fusobacterium nucleatum, Porphyromonas asaccharolytica, Parvimonas micra, Prevotella intermedia, Alistipes finegoldii* and *Thermanaerovibrio acidaminovorans*) enriched in CRC patients, which could serve as potential bacterial markers for the identification of CRC.^11,12^

Bacteria in the colon convert dietary fiber into SCFAs, which act as an energy source to the host, particularly in the case of butyric acid.^13,14^ In the absence of dietary polysaccharides, *Bacteroides thetaiotaomicron* rely on mucin glycans as an energy source, which results in a reduced colonic mucus layer.^15^ As compared to the small intestine (made up of a single mucus layer), the colon has two distinct mucus layers, i.e., outer (loose) and inner (tight) layers tethered to the epithelium.^16^ The inner tight mucus layer is rich in mucin-2 (MUC2), secreted from the goblet cells as a disulfide cross-linked network and less colonized by commensal microorganisms.^17^ The polysaccharide content, majorly *O*-linked glycans (80% of the mucin biomass) in the mucus layer, acts as an energy source for the gut bacteria.^18^ One study found genetic deletion of Muc2 (Muc2^−/-^) is associated with the development of colitis-associated CRC in mice. Further, Muc2^−/-^ mouse epithelium showed changes in mucosal thickening, increased proliferation, and superficial erosions in the distal colon due to reduced mucus layer thickness with altered microbiota.^19^ Also, both proximal and distal colons in Muc2^−/-^ mice are negative for the expression of mucins Muc5ac and Muc5b, while transient de novo expression of Muc6 was observed in the distal colon. The authors suggest that a small subset of goblet-like cells in the crypt base might be expressing Muc6 as a compensatory mechanism in Muc2^−/-^ mice.^19^

The symbiotic relationship between host and gut microbiota is important to maintain intestinal mucus layer homeostasis and prevents CRC. Hence, secretion of mucins by the goblet cells and degradation by the gut microbiota needs to be balanced.^20^ The present review summarizes the evidence in animal and human studies elucidating the different strategies including prebiotics, probiotics, and postbiotics (metabolites secreted by the gut microorganisms) on gut microbiota modulation, mucus composition, and mucins regulation.

## Interaction of diet and gut microbiota: CRC risk

CRC is the 3^rd^ leading cause of cancer-related deaths in the USA, with an estimated 1,49,500 new cases and 52,980 deaths in 2021.^21^ It is a multifactorial disease in which several genetic, environmental, and dietary factors are involved. Further, recent studies highlighted the central role of gut microbiota during the development of CRC. In the United States, around 50–60% of CRC incidents were estimated to be due to a change in lifestyle, especially diet;^22–24^ however, the exact mechanism by which dietary factors cause CRC remains unknown. Several studies signify that these factors are strongly related to gut microbiota during the development of CRC.^25^ Here, we are focusing only on dietary patterns that alter gut microbiota and their postbiotics role in CRC development, though the role of other factors has been extensively reviewed by others.^26–28^

According to epidemiologic studies, sporadic CRC can be associated with the diet.^29^ As shown in Figure 1, a Western-style diet (rich in fat or sucrose, red and processed meat, and low in fiber) alters gut microbiota, and can influence the integrity of the intestinal mucus layer; however, the association between diet, gut microbiota, and mucus layer remain unclear.^30^ Some of the studies delineating the role of dietary fibers in CRC have reported conflicting results.^31^ This is due to the source of fiber (cereals *vs*. fruits) or individuals having a different composition of gut microbiota, variation in the treatment duration, and heterogeneity in tumor subtypes.^32^ Recently, experts from World Cancer Research Fund and American Institute for Cancer Research in 2017 (https://www.wcrf.org/dietandcancer/colorectal-cancer) reported that consumption of red and processed meat (100 g/day, each) increases the risk of CRC, while intake of whole grains and dairy products (90 and 400 g/day) decreased the risk for CRC.^33^ Another study, switched mice from chow to a western diet for 28 days and showed an increase in the permeability of the intestine and a decrease in the thickness of the inner mucus layer (especially reducedMuc2 polymeric network). By contrast, supplementing mice with inulin fiber along with *Bifidobacterium longum* sufficiently restored mucus growth.^30^ The absence of dietary fiber in gnotobiotic mice (populated with known organisms) increases access of *Citrobacter rodentium* to the colonic mucus layer, damaging the mucosa and resulting in colitis and eventually CRC. The increase in *C. rodentium* results in higher expression of carbohydrate-active enzymes that in turn degrade mucins for their energy.^17^ In contrast to this, colonization of butyrate-producing bacterium *Butyrivibrio fibrisolvens* in the presence of a high fiber diet (2% cellulose and 6% fructo-oligosaccharide/inulin) protected gnotobiotic mice from CRC development.^34^

Interestingly, consumption of a western diet by Japanese migrants in Hawaii increased their risk of developing CRC, similar to the level observed in white individuals in the United States.^35^ This is due to the high intake of red meat associated with altered activities of type I and II carcinogen-metabolizing enzymes, resulting in the formation of heterocyclic aromatic amines (HAAs).^35^ These HAAs require activation by CYP1A2 and N-acetyltransferase before they can bind to the DNA.^36^ Another study examined the association between diet and gut microbiota in African Americans (AAs), prone to CRC risk, and native Africans (NAs), with low CRC risk.^37^ The study found that AAs consume more animal products such as meat, protein, saturated fat, and cholesterol compared with NAs, which was associated with higher colonic mucosal crypt proliferation and lower fecal Lactobacilli species.^37^

Similarly, the same group also investigated how diet (fat and fiber) and gut microbiota mediate CRC risk in AAs and NAs.^38^ When AAs switched to a high-fiber and low-fat diet for 2 weeks, they exhibited decreases in mucosal inflammation (CD3^+^ lymphocytes and CD68^+^ lamina propria macrophages) and secondary bile acid synthesis and increases in saccharolytic fermentation and butyrogenesis. These findings suggest consumption of less meat, fat and more carbohydrates and fiber might increase butyrogenesis, thereby mitigating CRC risk.^29,37,38^ Recently, *Fusobacterium nucleatum*-infected patients showed a lower risk of CRC upon intake of a prudent diet, which was rich in whole grains and dietary fiber.^39^ The increased amount of *F. nucleatum* in CRC tumor tissues was associated with poor survival,^40^ though the exact mechanism by which dietary fiber decreased the risk of CRC is not known. The proposed mechanism is that greater fermentation of carbohydrates present in the prudent diet alters the SCFAs composition, which results in a change in pH, longer transit time in the gut, and greater immune surveillance to inhibit the colonization of *F. nucleatum*.^38,39^ Overall, the presence of distinct bacteria populations that prevent mucus layer damage, inflammation, and associated CRC depends on the consumption of specific dietary fibers and phytochemicals, also.

Phytochemicals are secondary plant metabolites that constitute dietary fiber which are poorly metabolized in the upper gastro-intestinal tract and modulate the intestinal microbiota in colon such as *Akkermansia muciniphila*, resulting in prevention of intestinal inflammation in mice.^41,42^ Supplementation of *Akkermansia* in the high-fat diet-fed mice restores the lipopolysaccharide mediated gut permeability and leakage and preserves the intestinal mucus layer thickness.^43^ In addition to promoting anti-inflammation, treatment of polyphenols extracted from blueberries and olive oil also showed significant inhibition of growth in CRC cell lines (HCT-116 and HT-29) along with induction of cell cycle arrest, and apoptosis.^44,45^ Administration of phytochemical pterostilbene (structurally similar to resveratrol) decreased tumors in colon along with reduction of β-catenin and cyclin-D1 markers in chemically induced (Azoxymethane, AOM) CRC mice model.^46^ Further, pterostilbene also led to decrease in pro-inflammatory cytokines, tumor necrosis factor-alpha, interleukin (IL)-1beta and IL-4 in mucosa suggesting its potential role in CRC prevention.^46^ Taken together, phytochemicals and their microbial metabolites could be used as a complementary therapy against CRC while, its importance toward clinical trials depends on individual gut microbial composition.

## Amelioration of gut microbiota and mucins: pre and probiotics

**Figure 2.**
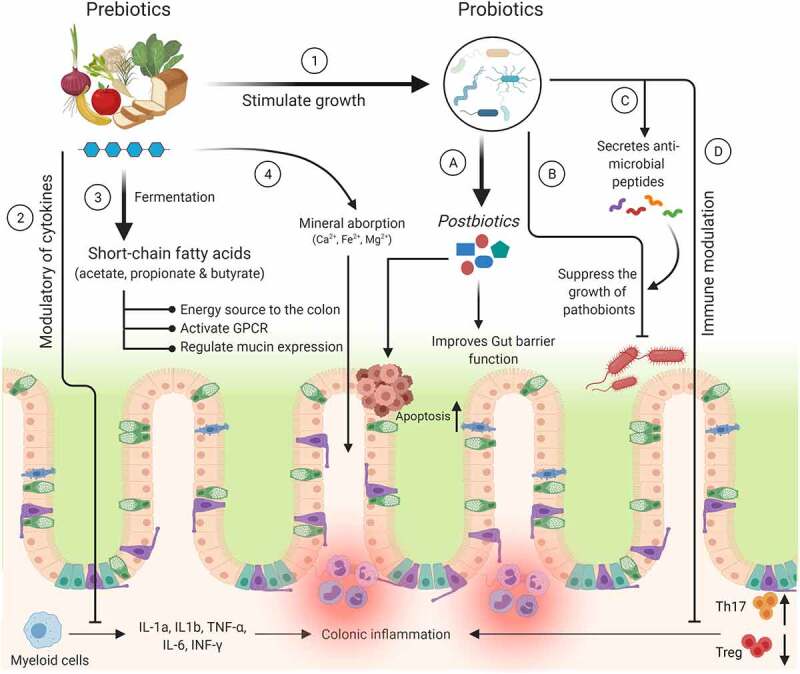
Mechanism(s) of action of prebiotics and probiotics. The health benefits of prebiotics on the host are involved with various mechanisms, *viz*., 1) Selectively stimulate the growth of beneficial probiotic organisms. 2) Modulate various cytokines to inhibit the secretion of pro-inflammatory markers. 3) & 4) Release SCFAs and increase the absorption of minerals. In case of probiotics: A) Mediate the secretion of metabolites from the prebiotics (e.g., fiber) or directly involved in the inhibition of CRC tumor growth by increasing apoptosis. B) Suppress the growth of harmful bacteria by reducing intestinal luminal pH. C) Secrete the anti-microbial peptides (bacteriocins and β-defensins) to inhibit the growth of pathobionts. D) Decrease colonic inflammation by reducing Th17 and increasing Treg cells

Modulation of gut microbiota by different interventions is needed to protect individuals at high risk of colitis and CRC. Various studies have shown that the inclusion of non-digestible carbohydrates in the diet called prebiotics improves gut microbiota. Prebiotics are “substrates that are selectively utilized by host microorganisms conferring health benefits to the host”.^47^ The concept of using prebiotics was first defined by Glenn Gibson and Marcel Roberfroid in 1995.^48^ Prebiotics are selectively fermented by probiotic microorganisms, leading to the production of SCFAs (acetate, propionate, and butyrate).^49^ Further, SCFAs have different modes of action, such as acting as an energy source to the colonocytes, regulating MUC2 expression for the intestinal barrier function, and activating G-protein-coupled receptor signaling to modulate immune function (Figure 2).^50^ In addition, SCFAs also participate in the activation of AIM2 and NLRP3 inflammasomes, stimulating interleukin-18 production that results in improved epithelial barrier function.^50–52^ Indeed, prebiotic supplementation decreased colonization of pathogens in human studies, potentially through SCFA action. The most common prebiotics are galacto-oligosaccharides (GOS) and fructose-oligosaccharides (FOS).^53^ GOS is composed of oligo-galactose and produced commercially from lactose by the enzyme β-galactosidase; however, FOS is naturally present in chicory root, onion, garlic, asparagus, and banana, as well as synthesized commercially.^54^ Here, we discuss the effect of GOS and FOS on the mucus layer functionalities, gut microbiota modulation, and CRC prevention.

Due to its β (1–4) glycoside linkage, GOS is not metabolized in the small intestine and reaches the colon, where it is degraded by the gut microbiota to release SCFAs, lactate, and several gases.^55^ It has been reported that GOS is involved in the modulation of the intestinal goblet cell and mucus barrier functions.^56^ In addition, incubation of GOS with LS174T cells stimulates goblet cells to produce mucus and also decreases the expression of genes involved in mucus layer permeability.^56^ Supplementation of GOS to germ-free rats resulted in reduced mucus distribution (neutral, acid, and sulfonated mucins) in the proximal colon compared with conventional rats. The reason might be due to more production of SCFAs by the microorganisms present in conventional rats.^57^ In another study, feeding of GOS (5%) to BALB/c mice for 4 weeks led to higher expression of intestinal mucosa-associated mucins (Muc2 and Muc4) at the transcript level as compared to control animals.^58^ Administration of GOS (derived from lactulose) for 20 weeks decreased colon tumor formation in a chemically (azoxymethane and dextran sodium sulfate) induced CRC rat model.^59^ Furthermore, metagenomic sequencing of colon gut microbiota revealed that GOS-treated animals had significantly reduced pro-inflammatory microorganisms and increased beneficial *Bifidobacterium* levels compared with control animals.^59^ Next, treatment of CRC line (LS174T) with GOS containing lactose (GOS-lac) resulted in a significant increase in the expression of MUC2 and its co-secreted molecule trefoil factor-3 (maintains mucus layer integrity) along with RELMβ (anti-parasitic activity); thus, GOS formulations may be effective in treating gut-associated disorders.^56^ Studies have shown that intestinal alkaline phosphatase (ALP) has a protective role in inflammatory disease and also prevents bacterial invasion by detoxifying lipopolysaccharide.^60^ Rats on a high-fat diet containing GOS showed elevated colonic ALP activity, improved intestinal barrier function, and greater microbial fermentation compared with rats without GOS, suggesting its importance in gut epithelial homeostasis.^61^

Another non-digestible carbohydrate is FOS and beneficial to the host health because of its protective role in intestinal microbiota.^48^ FOS have 2 to 7 fructose units with a β (2–1) linkage. Similar to GOS, FOS are not metabolized in the small intestine and reach the colon, where they stimulate endogenous bifidobacteria and lactobacilli species.^62^ FOS supplementation (60 g/kg body weight) for 35 days resulted in a decrease in the number of aberrant crypt foci in the colon of rats with chemically (1, 2-dimethylhydrazine) induced CRC.^63^ Similarly, in a genetically engineered Apc^Min/+^ mouse model, feeding with short-chain FOS (scFOS) delayed or reduced the development of adenoma tumors in the colon due to activation of anti-tumor immunity.^64^ Moreover, feeding of FOS (50% in the diet) to growing rats led to an increase in *Bifidobacterium* and *Lactobacillus* populations and Muc4 (involved in intestinal epithelial cell differentiation, renewal and lubrication) expression compared with untreated and aging rats, suggesting that consumption of FOS could be effective in modulating the microbiota in younger rats.^65^ Further, healthy human subjects ingesting FOS (12.5 g/day) showed a promising increase in fecal bifidobacteria and no effect on fecal total anaerobes, change in pH, nitroreductase, azoreductase, and β-glucuronidase activities, along with unchanged concentrations of bile acids and neutral sterols, which are involved in CRC progression.^66^ On the contrary, consumption of scFOS (10 g/day for 3 months) resulted in an increase in butyrate (acts as the principal energy source for colonocytes and increases mucin production) levels in feces of patients with different sized adenomas (<10 mm or larger in diameter).^67^ Overall, studies are focusing on the role of GOS and FOS in mucus production and prevention of intestinal metabolic disorders and CRC. However, the exact mode of action is not known. Therefore, supplementation of GOS/FOS in food could be a valuable strategy to treat these intestinal disorders and requires further investigation.

Probiotics are live microorganisms that, when administered in adequate amounts, confer a health benefit to the host.^68^ The mechanisms of probiotics are briefly summarized in Figure 2. Studies have shown that probiotic supplementation diminished symptoms in patients with inflammatory bowel diseases (IBDs) by enhancing the intestinal barrier at the mucosal surface.^69^ Oral administration of clinically tested VSL#3 probiotic cocktail (having Lactobacilli, Bifidobacteria, and Streptococci, 3 × 10^9^ cells) to rats for 7 days resulted in an increase in the mucin secretion by 60% in the colonic lumen and stimulated Muc2 and slight elevation of *Muc1* and *Muc3* gene expression upon exposed isolated rat colonic loops to the VSL#3 formula.^69^ Further, incubation of colonic epithelial cell line (LS174T) with conditioned media from live VSL#3 bacteria did not stimulate mucin secretion. However, lactobacilli secreted products in the secretome showed promising mucin secretion. The upregulation of mucins is likely due to an increase in the activity of already differentiated goblet cells as a strategy for allowing colonization of the microbiota.^69^ Similarly, potential probiotic *Lactobacillus rhamnosus* CNCM I-3690 (5 × 10^9^ cfu) promotes the expression of endogenous protease inhibitor Kazal-type 4 (Spink4) and amino acid transporter SLC7A7 in specific pathogen-free C57BL/6 mice.^70^ The upregulation of Spink4 inhibits the proteolytic degradation of epithelial and mucosal tissue in IBDs, while SLC7A7 increases the availability of the amino acids for the growth of intestinal epithelial cells.^71^ Additionally, supplementation of CNCM I-3690 increased the production of IL-10 in the colon tissue to protect the mucus barrier by suppressing protein misfolding and ER stress in goblet cells. These results suggest that the administration of CNCM I-3690 could be a promising strategy to prevent gut barrier dysfunction.^70^

## Mucin regulation and intestinal barrier function by gut microbiota metabolites: postbiotics

**Figure 3.**
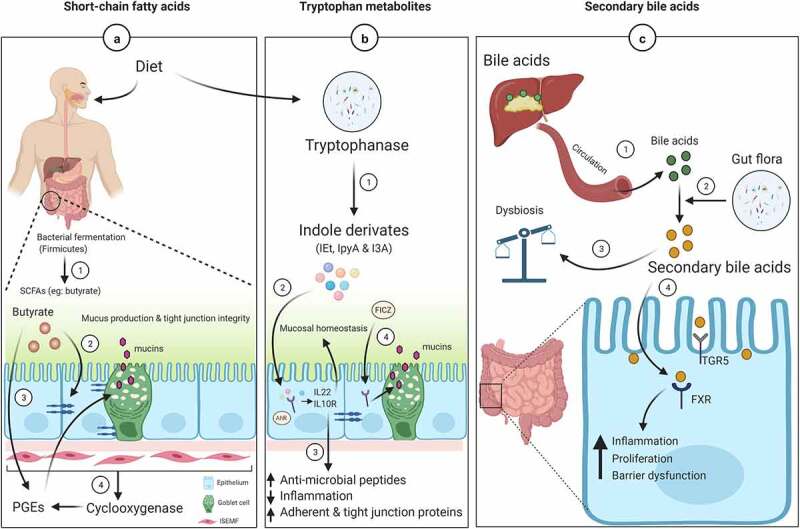
Mucin regulation and intestinal barrier function by gut microbiota metabolites

Interaction of gut microbiota and the host is crucial for maintaining gut homeostasis. The crosstalk between the microbiota and host occurs via metabolites (*postbiotics*), which are released via fermentation from non-digestible compounds in the colon.^72^ The metabolites or products derived from the microorganisms are effective in treating many diseases,^73^ and these postbiotics are currently being used in clinical studies (Table 1). Below we describe the bacterial metabolites and their role in mucin regulation and intestinal mucus layer formation (Figure 3).

**Table 1.** Clinical Studies reporting interventions with postbiotics products in infants, toddlers, and adults

S.No.	Clinical trial number	Postbiotics	Individual (eligibility criteria)	Participants (*N*)	Status	Major study details
1.	NCT04745455	Cow’s milk based infant formula having prebiotics, probiotics and postbiotics	Infants (up to 84 days)	30	Recruiting	Investigating about gastrointestinal tolerance.
2.	NCT04151823	Postbiotics from Lactobacillus paracasei CNCM I-5220 and SMART D3 MATRIX contains vitamin D3	Childhood obesity (6 to 14 years)	30	Recruiting	Determine the alterations in the gut microbiota composition and short-chain production.
3.	NCT04042454	Cow’s milk-based infant formula having the bean gum prebiotic oligosaccharides and postbiotics	Healthy infants (up to 9 weeks)	100	Recruiting	Evaluating the safety and tolerance of formula in infants with regurgitation.
4.	NCT04324749	Roasted peanuts and peanut butter	Adult (18 to 32 years)	90	Completed	Identification and quantification of polyphenols, short-chain fatty acids and evaluation of the gut microbiota profile.
5.	NCT04267731	Bifidobacterium breve extract (VMK223) and cellulose	Adult (50 to 65 years)	30	Active, not recruiting	Gut health, inflammation, and aging process.
6.	NCT04151758	Docosahexaenoic acid supplementation	Childhood obesity (6 to 14 years)	30	Recruiting	Evaluating gut microbiota composition and function.
7.	NCT04908644	Fermented soybean extract MicrSoy-20(MS-20)	Adult (20 to 65 years)	40	Not yet recruiting	Gut microbiota alterations in ulcerative colitis.

(A) SCFAs modulate intestinal epithelium. (1) Fermentation of non-digestible carbohydrates by bacteria produces SCFAs (acetate, butyrate, and propionate) in the colon. (2) Butyrate is the main energy source for the colon, and it regulates epithelial junction proteins. (3) In addition, it is also responsible to produce MUC2 along with PGEs. (4) The release of PGEs by cyclooxygenase, which is expressed by the intestinal sub-epithelial myofibroblasts (located below the epithelium) also maintains the mucus layer function.(B) Tryptophan (Trp) metabolites maintain mucosal homeostasis. (1) Gut microbial enzyme tryptophanase degrades Trp into indole derivatives such as indole-3-ethanol-IEt, indole-3-pyruvate-IPyA, and indole-3-aldehyde-I3A. (2) These derivatives bind to aryl hydrocarbon (AhR), stimulating the release of IL-22 and IL-10. (3) Further, these cytokines mediate upregulation of anti-microbial peptides and adhesion (E-cadherin and β-catenin) and tight junction (ZO-1 and Occludin) proteins and decrease inflammation to maintain intestinal homeostasis. (4) Similar effects were also observed with the photoproduct of Trp FICZ upon binding to AhR.(C) Dysbiosis is associated with secondary bile acids. (1) After synthesis from cholesterol in the liver, bile acids are released into circulation as a bile juice for digestion. In the small intestine, 95% of bile is reabsorbed; however, (2) the remaining 5% is metabolized by the bacteria in the colon to form secondary bile acids. (3) Secondary bile acids have important functions in gut dysbiosis and (4) regulate several physiological functions after binding to a G-coupled receptor (TGR5) and nuclear receptor (FXR).

### Short-chain fatty acids

SCFAs (acetate, propionate, and butyrate) are produced via bacterial fermentation^13,14^ and help to create a barrier between the gut epithelium and the external environment. Butyrate is mainly produced by Firmicutes bacteria and is the preferred metabolic substrate for colon epithelial cells, whereas acetate and propionate are predominantly produced by Bacteroidetes.^72^ The role of butyrate in regulating mucin expression was evaluated in the HT29-Cl.16E CRC cell line (derived from HT-29) cultured in glucose-enriched media. Treatment with butyrate (2 or 5 mM) for 24 hours significantly increased the expression of MUC3 and MUC5B but decreased MUC5AC levels and had no effect on MUC2 expression in the presence of a glucose-high medium. Similar results were observed in the absence of glucose and the presence of butyrate, except in this case, MUC5AC and MUC2 levels were higher. These effects were specific to butyrate and not observed with acetate and propionate treatments, suggesting butyrate regulates mucin expression because it is a major energy source for the colonocytes.^74^ Contrarily, both butyrate (1 mM) and propionate (1–15 mM) induced increased expression of *MUC2* at the transcript levels in LS174T cells. MUC2 promoter analysis suggested that an active butyrate-responsive region containing an AP1 (c-Fos/c-Jun) *cis*-element is required for the activation of MUC2 via acetylation and methylation of histones.^75^ Further, an investigation of the effect of butyrate on mucin secretion in LS174T CRC cells revealed the influence of beneficial probiotics (*Lactobacillus* and *Bifidobacterium*) species.^76^ Treatment of LS174T cells with butyrate (6 or 9 mM) led to increased mucin protein content, which enhanced adherence of probiotic strains, thereby inhibiting pathogenic *Escherichia coli* (*E.coli*) attachment. Also, butyrate increased the expression MUC3, MUC4, and MUC12, while having no effect on MUC2 levels. The up-regulation of specific mucin genes was accompanied by upregulation of genes involved in mitogen-activated protein kinase signaling, which are associated with increased cell mass and cellular growth.^76^ Rectal administration of butyrate (140 mM) resulted in decreased inflammation in mice challenged with *Citrobacter rodentium*. In addition, the expression of *IL-10, Tgf-β*, and *Muc2* was elevated along with pathogen clearance genes (*IL17A* and *IL1β*) and genes involved in intestinal barrier repair and restoration (*Relm, Tff3* and *Myd88*).^77^

The intestinal mucus layer protects gut epithelial cells from various foreign objects by secreting mucus. This secretion can be influenced by physiological and immune modulators like prostaglandins (PGEs). Studies suggest that intestinal sub-epithelial myofibroblasts (ISEMF, present below the epithelium) regulate epithelial cell functions such as proliferation, differentiation, secretion, and motility.^78^ During tissue injury or inflammation, ISEMF express cyclooxygenases (COX), responsible for the synthesis of PGEs (orchestrates inflammatory response) (Figure 3(a)). Inhibition of COX through non-steroidal anti-inflammatory drugs results in intestinal lesions such as intestinal bleeding, perforation, ulcers, inflammation, and strictures requiring surgery.^79^ Therefore, analogs of PGEs play an important role in protecting the intestinal mucus barrier. Co-culturing CRC cell lines (LS174T and T84) directly with intestinal myofibroblasts (CCD-18Co) in the presence of SCFAs (0.025–4.0 mM) as well PGE1or PGE2 (0.01–100 ng/ml) for 24 hours led to increased expression of *MUC2* as compared to a monolayer cell culture system.^78^

As stated above, dysbiosis is associated with several diseases, including IBDs. The levels of butyrate were significantly reduced in the stools of Japanese patients with UC and CD as compared to healthy individuals. In contrast, UC patients had higher levels of mucin *O*-glycans in their stools, likely due to the altered gut microbiota, which utilize *O*-glycans less efficiently. These findings suggest that O-glucans serve as an endogenous fermentation source for butyrate-producing bacteria.^80^ Therefore, understanding the role of SCFAs, especially butyrate, in modulating mucus layer synthesis is crucial in the management of gastrointestinal diseases.

### Tryptophan metabolite

Among the bacterial metabolites, tryptophan (Trp) is one of the essential amino acids supplied through the diet and plays an important role in intestinal permeability.^81^ Most of the Trp (95%) is catabolized by the host enzyme indoleamin 2,3-dioxygenase 1 (IDO1) to produce kynurenine. In addition, some of the Trp (4–6%) is also degraded by the gut microorganisms (via tryptophanase) to produce indole derivatives (indole-3-ethanol-IEt, indole-3-pyruvate-IPyA, and indole-3-aldehyde-I3A and 3-indole-propionic acid, IPA), serotonin and tryptamine. These metabolites regulate intestinal barrier function by upregulating aryl hydrocarbon receptor (AhR, transcription factor) in mice and humans.^82,83^ Trp metabolites are thought to be produced by *Lactobacillus reuteri* and *Clostridium sporogenes* in mice and the human gut. The activation of AhR by indole derivatives results in IL-22 as well as IL-10 R secretion to stimulate anti-microbial peptide expression, epithelial cell proliferation, tight junction protein expression, and mucin production while inhibiting lipopolysaccharide-induced inflammation.^81,84^ The tryptophan metabolite signal also occurs through pregnane X receptor (PXR) to regulate intestinal integrity.^85^ Mice deficient in PXR showed a leaky gut pathology that resulted in decreased Muc2 expression, suggesting Ahr and PXR pathways are necessary for the expression of *Muc2*.^85^ Feeding rats a high-fat diet damages the epithelial barrier, which is reversed by IPA treatment via increased *Muc2* and *Muc4* expression, which strengthen the mucosal layer.^86,87^ In addition, IPA also up-regulates MUC2 expression in a colonic spheroid culture system.^88^ A combination of Trp and *Lactobacillus plantarum* KLDS 1.0386 (high Trp-metabolic activity) treatment improved the epithelial and mucus barrier by decreasing the expression of pro-inflammatory cytokines (TNF-α, IL-1β, and IL-6) in adextran sodium sulfate (DSS)-induced colitis mouse model.^89^ The combination also increased the expression of tight-junction proteins (ZO-1, claudin, and occluding), mucins (*Muc1* and *Muc2*), and Trp metabolite (IAA) in the colon. IAA upregulates *AHR* transcript levels, resulting in activation of the IL-22/STAT3 signaling pathway.^89^

Indole derivatives (IEt, IPyA, and I3A) maintain the apical junctional complex (AJC), which comprises tight junction (ZO1 and occludin) and adherent junction (E-cadherin and β-catenin) proteins.^90^ A recent study demonstrated that diets rich in Trp ameliorates DSS-induced colitis in mice by supplying indole derivatives, which activate the AhR receptor. The mechanism by which these derivatives modulate AJC is by inhibiting activation of actin regulatory (ezrin and non-muscle myosin II) proteins, thereby decreasing intestinal permeability^83^ (Figure 3(b)). Similarly, a photoproduct of Trp (FICZ) activates AhR, resulting in enhanced MUC2 expression and goblet cell proliferation, and inhibition of bacterial infiltration in DSS-induced colitis mice. This finding suggests the upregulation of MUC2 by FICZ might be due to AhR-ERK signaling.^91^ Therefore, a protein diet rich in Trp could be beneficial to the host by improving intestinal defenses.

### Secondary bile acids

Metabolites from the gut microbiota as well as host-derived molecules play an important role in human metabolism. For example, bile acids (BAs) are synthesized from cholesterol in the host liver by the enzyme cholesterol 7 alpha-hydroxylase (CYP7A1) and subsequently conjugated to glycine or taurine to form bile salts.^92^ The conjugated BAs are reabsorbed in the gut via apical bile salt transporters, whereas 5–10% of BAs are not reabsorbed and instead are metabolized by the gut microbiota to produce secondary BAs (deoxycholic and lithocholic acids).^92^ These secondary BAs act as signaling molecules via nuclear farnesoid X receptor (FXR) and G-protein-coupled receptor (TGR5) to regulate many biological functions^93^ (Figure 3(c)). A diet containing high-fat increases the secondary BAs, mostly deoxycholic acid (DCA), to induce dysbiosis by inhibiting the growth of *Clostridium perfringens, Bacteroides fragilis, Lactobacilli*, and *Bifidobacteria*, which are associated with intestinal tumors.^94^ Supplementation with DCA (0–100 μM) induced higher expression of *MUC2* and E-cadherin, while markedly decreasing the expression of tumor invasion and migratory molecules, i.e., Snail and MMP9, in gastric cancer cells.^95^ The upregulation of MUC2 is useful for the prediction of gastric cancer and its prognosis.^95^ Similar results were also observed with CRC cell line (H3) where DCA regulates *MUC2* expression via multiple pathways (EGFR/PKC/Ras/Raf-1/MEK1/ERK/CREB, PI3/Akt/IkappaB/NF-kappaB).^96^

Transfer of fecal microbiota from DCA-fed animals to Apc^min/+^ mice along with the antibiotic streptomycin resulted in low-grade inflammation and increased tumor burden due to gut microbiota alteration.^97^ In addition, DCA-fed animals also showed a reduction in the expression of E-cadherin and up-regulation of β‐catenin levels. The reduction of E-cadherin might be due to alteration of *Fusobacterium nucleatum* adhesin FadA, which is required for attaching E-cadherin on epithelial cells to mediate Wnt/β‐catenin signaling.^97,98^ In a later study, cholic acid-fed Apc^min/+^ mice showed an increase in the mucin-degrading bacteria (Akkermansia and Bacteroides) and a decrease in SCFAs and MUC2 expression, resulting in cancer progression via STAT3 signaling.^99^ Therefore, understanding the association between BAs and microbiota on intestinal epithelial cells is important in mucosal physiology.

## Fecal microbiota transplantation

Recently, fecal microbiota transplantation (FMT) has gained importance for treating gut dysbiosis.^100^ FMT involves transferring stools (in capsule form) from healthy individuals to patients with disease via an endoscope and nasoenteric tube.^101^ The overall goal of FMT is to establish a new microbiota community in the gut to treat IBD, autoimmune disorders and metabolic diseases.^102^ In the case of *Clostridium difficile* infection, which causes inflammation in the colon, FMT is used as a second-line treatment with a 92% success rate in clinical studies.^103,104^ FMT donors are mainly from two sources: patient-directed donors and universal donors via stool banks. Patient-directed donors are usually family members of the patient and are less frequently used because of cost.^105^ However, universal donors (healthy volunteers) from stool banks have been widely used for FMT due to extensive screening procedures (https://www.openbiome.org/safety). Feces from CRC patients fed to conventional (treated with AOM) and germ-free mice showed high-grade dysplasia with increase in macroscopic polyps, tumor cell proliferation, and inflammation compared to healthy stool-fed mice.^106^ Transfer of microbiota from wild mice to laboratory mice also showed resistance to chemically (AOM/DSS) induced CRC and improvement in the inflammation.^107^ In addition to mouse models, application of FMT to piglets infected with *E. coli* K88 (to cause epithelial injury) enhanced beneficial bacteria, *Lactobacillus* and *Succinivibrio*, along with an increase in metabolites and metabolic pathways (branched-chain amino acids and linoleic acid metabolism).^108^ Further, FMT modulated gut barrier function by decreasing intestinal permeability and increasing mucin (Muc2) and tight junction proteins (ZO-1 and occludin) in the piglets.^108^ In a recent study, administration of feces from CRC patients in Apc^Min/+^ mice led to an increase in proliferation, pro-inflammatory cytokines, and decreased apoptosis in tumors cells.^109^ Additionally, these mice also exhibited diminished gut barrier function tight junction proteins, ZO-1, occludin, and claudin3 and activation of Wnt signaling pathway.^109^ In the future, FMT could be used as microbiotic therapy to treat various colonic diseases, and further clinical evidence is needed to establish its safety.

## Conclusion and perspective

The gut microbiota and the host epithelial mucus layer are key players in maintaining and protecting the large intestine. Mucins, which are major components of mucus, not only act as a defense against pathogens but also serve as an energy source for the microorganisms. In disease states such as IBDs, CRC, and metabolic diseases, metabolites secreted from pathogenic bacteria reduce the thickness of the intestinal mucus layer. Therefore, researchers are focusing on ways to improve the gut barrier function and host health by various treatment strategies. Mostly, nutritional interventions (prebiotics, probiotics, and both) seems to improve the mucus layer by modulating the gut microbiota, and more studies are needed to establish their effective dose and safety concerns. Additional studies on the degradation of prebiotic fibers by specific probiotic microorganisms are needed to understand their mechanism of action in intestinal homeostasis. Similarly, metabolites produced during bacterial fermentation and their secondary metabolites facilitate the synthesis and regulation of mucins. Further research is needed to understand how dietary patterns regulate the mucins for mucus layer formation and modulation of gut microbiota, along with how FMT can be utilized in human health and disease. Below some of the fundamental questions that remain to be addressed are briefly listed below:

- Can we modulation the gut-microbiota by prebiotics, probiotics, or both to treat inflammatory bowel diseases and colorectal cancer (CRC) patients?
- How these dietary factors were effective in intestinal layer functionalities and improving gut-microbiota?
- What the molecular mechanism(s) are involved in the regulation of mucins by postbiotics?
- Is fecal microbiota transplantation being an effective strategy to restore the dysbiosis associated with CRC patients?
- Can we use tumor (CRC) associated bacteria for pre-clinical and clinical studies as diagnostic or prognostic markers?
